# Tyrosine kinase inhibitors in first-line treatment of advanced NSCLC with epidermal growth factor receptor mutations: Real-world data from Vietnam

**DOI:** 10.32604/or.2025.061905

**Published:** 2025-06-26

**Authors:** KHANH TOAN NGUYEN, THI HUONG PHAM, VAN LAM NGO, VAN TUAN BUI, VAN NHAT NGUYEN, THI PHUONG THAO NGUYEN, THI KHANH HA NGUYEN, THI THUY VAN NGUYEN

**Affiliations:** Department of Medical Oncology 2, Nghe An Oncology Hospital, Vinh City, 43000, Vietnam

**Keywords:** Non-small-cell lung cancer, EGFR, Osimertinib, Afatinib, Erlotinib, Gefitinib

## Abstract

**Aims:**

The study aimed to evaluate the effectiveness and adverse events of tyrosine kinase inhibitors (TKIs) in the first-line treatment of *advanced non*-*small cell lung cancer (NSCLC)* with epidermal growth factor receptor (EGFR) mutations.

**Methods:**

A retrospective study on advanced NSCLC patients with EGFR mutations treated with TKIs as a first-line therapy at Nghe An Oncology Hospital, Vietnam between January 2017 and August 2023. The primary endpoints included objective response rate, progression-free survival, and tolerability. The secondary endpoint was overall survival.

**Results:**

A total of 211 patients received first-line treatment with Erlotinib (n = 74), Gefitinib (n = 85), Afatinib (n = 34) or Osimertinib (n = 18). The overall response rate was 76.7%, with Osimertinib at 83.4%, Afatinib at 73.6%, Erlotinib at 77.1%, and Gefitinib at 76.5%. The median progression-free survival in the Gefitinib group was 12.2 months (95% CI: 11.1–13.2), 13.4 months (95% CI: 10.6–16.2) in the Erlotinib group, 18.4 months (95% CI: 10.1–26.8) in the Afatinib group and 25.3 months in the Osimertinib group (*p* = 0.001). The median overall survival was 21.8 months (95% Cl: 15.0–28.4) in the Gefitinib group, 30 months (95% Cl: 19.1–40.9) in the Erlotinib group (*p* = 0.154). Most drug-related adverse events were grade 1 or 2. Diarrhea was the most frequent adverse event in the Afatinib group at 44.1%; rash was most common in the Erlotinib group at 60.8%; paronychia (31.8%), and interstitial lung disease (3.5%) were most frequent in the Gefitinib group.

**Conclusion:**

The TKIs as first-line therapies for advanced NSCLC patients with EGFR mutated are highly effective, prolong survival, and are well tolerated.

## Introduction

In Vietnam, there were 24,426 new cases of lung cancer and 22,597 related deaths in 2022, placing it as the third highest in terms of incidence and second highest in mortality [1]. Some studies conducted in Vietnam showed that 70% of lung cancer patients were diagnosed with stages III or IV [2]. The main treatment options at this stage include chemotherapy, targeted therapy, and immunotherapy.

Since the 2000s, several genetic mutations identified in non-small cell lung cancer (NSCLC) have significantly changed patients’ prognosis [3–5]. The benefits of epidermal growth factor receptor-tyrosine kinase inhibitors (EGFR-TKIs) in improving response rates, prolonging survival, and limiting toxicity have been demonstrated in many phase III, multi-center clinical trials around the world, such as OPTIMAL [6], LUX-Lung 3 [7], LUX-Lung 6 [8], and FLAURA [9]. Consequently, these drugs have been recommended in international clinical practice guidelines, such as the NCCN [10] and the ESMO [11] as the first-line treatment of advanced NSCLC with EGFR mutations. EGFR mutations are divided into two groups [12]. The common mutation group includes exon 19 deletions and exon 21 L858R mutation, accounting for about 85%, and is generally highly responsive to TKIs [13]. Other mutations (S768I, G719X, and L861Q) are rare mutations, accounting for 10%–20% of mutations in NSCLC [14]. There has not been much research on the effectiveness of TKIs in this group. Some studies show that second- and third-generation TKIs (Afatinib and Osimertinib) are effective in rare mutation groups [15,16]. In Vietnam, Erlotinib, Gefitinib, and Afatinib are covered 50% by Vietnamese health insurance; Osimertinib is currently not covered by insurance; however, there is a program to support part of the cost by the Ministry of Health offering a buy-one-get-one-free deal.

Clinical trials have strict selection criteria and are usually conducted on only a specific group of patients, providing scientific evidence on the effectiveness of drugs. In addition, real-world data is collected from actual patients with various clinical characteristics [17]. Although there are many disadvantages, such as confounding factors and difficulties in controlling data quality, real-life data provide information on the effectiveness as well as the actual clinical benefits of drugs at a low cost. Currently, real-world studies on EGFR-TKIs published worldwide reinforce the evidence from clinical trials [17–19]. However, most of these studies evaluated the effectiveness of individual TKI types or compared two generations of TKIs, with limited real-world data comparing all three generations. In Vietnam, no studies have compared the effectiveness of different generations of TKIs as first-line treatments for metastatic NSCLC. In addition, there are no specific guidelines for selecting the type of medication for different patient populations. The Nghe An Oncology Hospital is a specialized cancer treatment center in the North Central region of Vietnam. The hospital has a planned capacity of 1200 beds and provides outpatient care for over 15,000 patients, with an average inpatient count ranging from 1100 to 1200 individuals. Therefore, we conducted this study to evaluate the effectiveness and adverse events (AEs) of TKIs as a first-line treatment for advanced NSCLC with EGFR mutations at Nghe An Oncology Hospital, Vietnam.

## Materials and Methods

### Study design

This study adhered to the guidelines of the Declaration of Helsinki. A retrospective study on 211 advanced NSCLC patients with positive EGFR mutation test results, treated as first line with EGFR–TKIs between 1/2017 and 8/2023 at Nghe An Oncology Hospital. The study protocol received approval from the Nghe An Oncology Hospital’s Research Ethics Committee. This approval was issued under decision number 784/QĐ-BVUB on 17 March 2023, by the Director of Nghe An Oncology Hospital.

### Study population

Inclusion Criteria: Patients aged ≥18 years with advanced stage (stage IIIB, IIIC without an indication for chemoradiotherapy or refusal of chemoradiotherapy, or stage IV). All patients had to harbor EGFR mutations associated with sensitivity to EGFR-TKIs (common mutations include exon 19 deletion and exon 21 L858R, uncommon mutations encompassing rare mutations and compound mutations) and should not have received any prior systemic therapy.

Exclusion Criteria: Patients had severe liver and/or renal dysfunction (Child-Pugh class C cirrhosis, glomerular filtration rate <15 mL/min), or had other malignancies in history.

All patients in the study were asked for their consent, and they agreed to participate in the study. They were notified of their complete right to withdraw from the study at any time without threats or disadvantages. Additional informed consent was obtained from all individual participants for whom identifying information is included in this article.

### Data collection

Data were collected with a cutoff date of August 30, 2023. To minimize bias, uniform criteria for patient selection and assessment, such as performance status (PS) score and response according to RECITS 1.1, were established. Two independent teams led by authors P.T.H. and N.V.L. conducted data collection. Any discrepancies or unclear cases in the data were reviewed and resolved through discussion within the research team. Principal investigator N.K.T. made the final decision.

### Treatment protocol

Eligible patients were treated with Erlotinib (150 mg/day) (Tarceva, Roche, Basel, Switzerland) or Gefitinib (250 mg/day) (Iressa, AstraZeneca, Cambridge, UK) or Osimertinib (80 mg/day) (Tagrisso, AstraZeneca, Cambridge, UK) or Afatinib (40/30/20 mg/day) (Giotrif, Boehringer Ingelheim, Ingelheim, Germany), the choice of TKI and dosage adjustments were made by the treating physician based on age and Eastern Cooperative Oncology Group (ECOG) status. Patients were assessed every three months or upon the appearance of abnormal symptoms. In cases of disease progression, patients with a detected Thr790Met (T790M) mutation were treated with osimertinib, whereas those with other mutations received targeted therapy. Patients without drug-resistance mutations or histological transformations were managed with chemotherapy or palliative care.

### Molecular testing

EGFR mutations are detected in tissue biopsies or blood/pleural fluid samples, mainly by real-time polymerase chain reaction AmoyDx EGFR 29 Mutation Detection Kit (Amoy Diagnostics, Haicang, Xiamen, China) or EGFR Mutation Analysis Kit (Entro Gen, Los Angeles, CA, USA) or next-generation sequencing (NextSeq, Illumina, San Diego, CA, USA).

### Outcome measures

Treatment response was evaluated based on the RECIST version 1.1 criteria. The overall response rate (ORR) included complete response (CR) and partial response (PR), whereas the disease control rate (DCR) included CR, PR, and stable disease (SD). Progression-free survival (PFS) was defined as the duration from the start of treatment to disease progression or death. For patients who were alive at the time of assessment or were lost to follow-up, PFS was evaluated by censoring. Overall survival (OS) is the time from treatment initiation to death. Patients who could not be contacted or were still living at the time of assessment were censored. AEs were collected and categorized according to the Common Terminology Criteria for Adverse Events (CTCAE) version 5.0.

### Statistical analysis

Data was analyzed using SPSS software version 20.0 (IBM Corporation, New York, NY, USA). Descriptive statistics are presented as frequencies and percentages for categorical variables and as medians with interquartile ranges (IQR) for continuous variables. Baseline characteristics, treatment responses, and safety outcomes were compared between groups using the chi-square test, Fisher’s exact test, or Wilcoxon rank-sum test, as appropriate. Statistical significance was set at *p* < 0.05. significant.

Survival curves were estimated using the Kaplan-Meier method. Factors influencing PFS in the study groups were analyzed using the Cox model. The Cox model included a term for study group assignments and additional terms for prespecified clinical variables.

The consistency of treatment effects on efficacy outcomes across prespecified subgroups was assessed using interaction tests between the treatment and reference groups within the Cox models.

### Research ethics

Research data are only collected when patients agree to participate in the study. All patient information is confidential and used only for research purposes. Our study is conducted and published honestly, independently, and without receiving funding from any organization or individual.

## Results

### Patients and treatment

**Figure 1:**
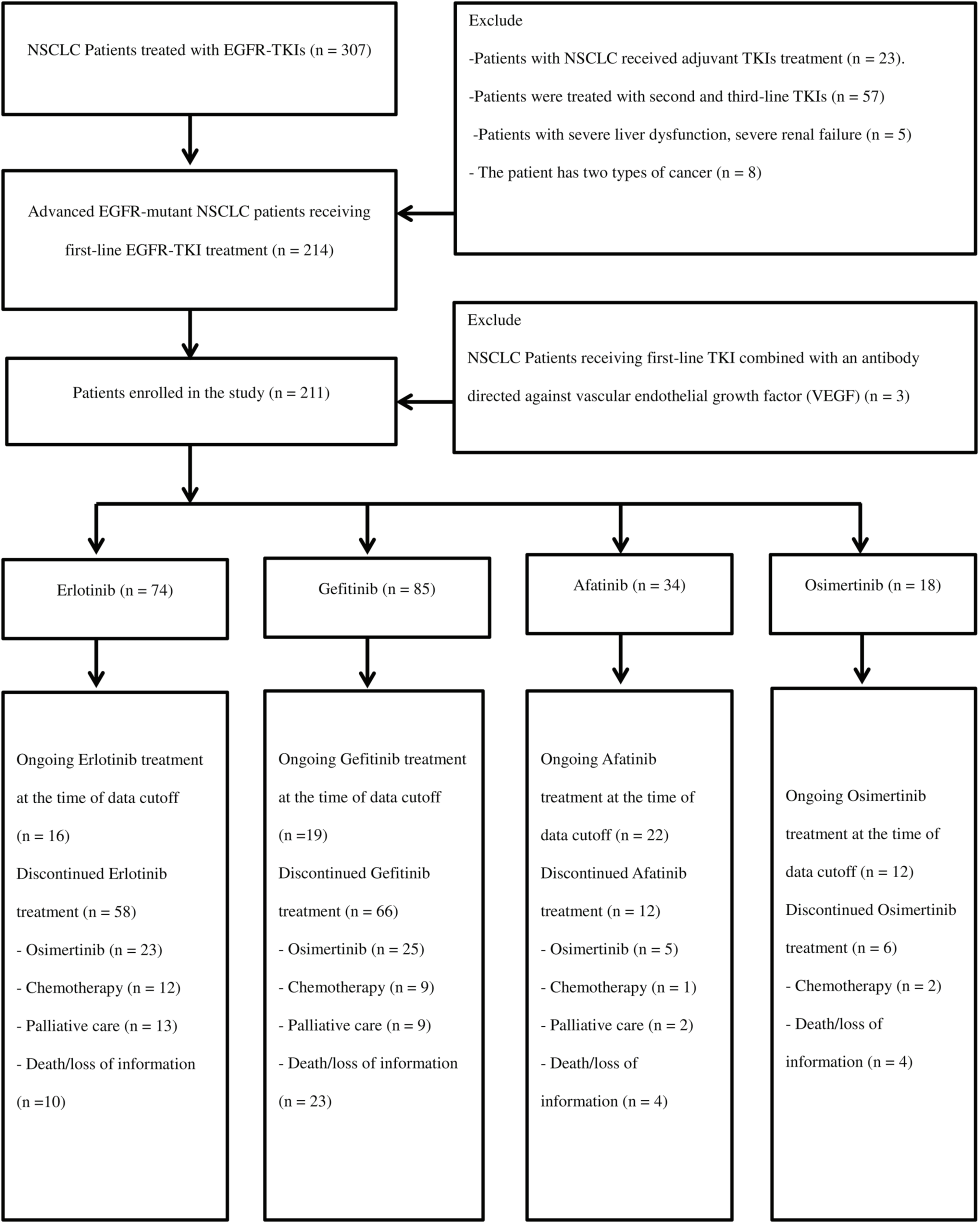
The flowchart of patient collection.

211 patients were included in the study. The patient selection process is shown in Fig. 1. The age groups under 65 and 65 years being quite similar in the Erlotinib and Gefitinib treatment arms (56.8% and 43.2% *vs*. 47.1% and 52.9%, respectively); and the percentage of patients aged ≥65 in the Afatinib and Osimertinib arms was higher, at 64.7% and 61.1%, respectively (*p* = 0.165). The percentage of non-smoking patients in the Gefitinib group was higher than in the Erlotinib (69.4% *vs*. 64.9%), Osimertinib (55.6%), and Afatinib (44.1%) groups (*p* = 0.066).

Bone, pleural-pericardial, CNS, and contralateral lung metastases were the most common, accounting for 46.9%, 46.4%, 27.0%, and 26.5% of cases, respectively. The proportion of brain metastases in the Osimertinib group was the highest at 38.9%, compared to that in the Gefitinib, Erlotinib, and Afatinib groups at 28.4%, 25.9%, and 20.6%, respectively (*p* = 0.547). The proportion of patients who received whole-brain radiation was higher in the Osimertinib group (16.7%) than in the Erlotinib (1.4%), Gefitinib (3.5%), and Afatinib (2.9%) groups (*p* = 0.024). Adenocarcinoma was predominant in 97.6% of the patients, while two patients had squamous cell carcinoma (0.9%). The rate of the common EGFR mutations was 93.8%. Thirteen patients (6.2%) had uncommon EGFR mutations, with the majority receiving Afatinib (6 patients, 17.6%) and Osimertinib (4 patients, 22.2%). The common EGFR mutations include Del 19 and L858R, with frequencies of 66.2% and 33.8% in the Erlotinib group, 63.5% and 32.9% in the Gefitinib group, 50% and 32.4% in the Afatinib group, and 66.7% and 11.1% in the Osimertinib group, respectively (*p* = 0.001). The characteristics of the patients are summarized in Table 1.

**Table 1:** Demographic and clinical characteristics of the patients

Characteristic		Erlotinib (n = 74) n (%)	Gefitinib (n = 85) n (%)	Afatinib (n = 34) n (%)	Osimertinib (n = 18) n (%)	Total n (%)	*p*-value
**Age**	<65	42 (56.8)	40 (47.1)	12 (35.3)	7 (38.9)	101 (47.9)	0.165
	≥65	32 (43.2)	45 (52.9)	22 (64.7)	11 (61.1)	110 (52.1)	
	Median	62.3 (30–79)	64.3 (37–87)	68.1 (40–86)	67.4 (50–91)	64.5 (30–91)	
**Sex**	Male	26 (35.1)	33 (38.8)	21 (61.8)	8 (44.4)	88 (41.7)	0.063
	Female	48 (64.9)	52 (61.2)	13 (38.2)	10 (55.6)	123 (58.3)	
**Smoking status**	Smoker	26 (35.1)	26 (30.6)	19 (55.9)	8 (44.4)	79 (37.4)	0.066
	Nonsmoker	48 (64.9)	59 (69.4)	15 (44.1)	10 (55.6)	132 (62.6)	
**ECOG PS**	PS < 2	53 (71.6)	62 (72.9)	30 (88.2)	11 (61.1)	156 (73.9)	0.145
	PS ≥ 2	21 (28.4)	23 (27.1)	4 (11.8)	7 (38.9)	55 (26.1)	
**Metastases**	Contralateral lung	22 (29.7)	20 (23.5)	11 (32.4)	3 (16.7)	56 (26.5)	0.518
	Pleural, pericardial	32 (43.2)	42 (49.4)	12 (35.3)	12 (66.7)	98 (46.4)	0.153
	Central nervous system	21 (28.4)	22 (25.9)	7 (20.6)	7 (38.9)	57 (27)	0.547
	Bone	43 (58.1)	38 (44.7)	12 (35.3)	6 (33.3)	99 (46.9)	0.07
	Liver	5 (6.8)	6 (7.1)	2 (5.9)	2 (11.1)	15(7.1)	0.913
**Histologic type**	Adenocarcinoma	73 (98.6)	82 (96.5)	33 (97.1)	18 (100)	206 (97.6)	0.726
	Squamous	0 (0)	1 (1.2)	1 (2.9)	0 (0)	2 (0.9)	
	Other*	1 (1.4)	2 (2.4)	0 (0)	0 (0)	3 (1.5)	
**EGFR mutation type**	*Del 19*	49 (66.2)	54 (63.5)	17 (50)	12 (66.7)	132 (62.5)	0.001
	*L858R*	25 (33.8)	28 (32.9)	11 (32.4)	2 (11.1)	66 (31.3)	
	Uncommon types**	0 (0)	3 (3.5)	6 (17.6)	4 (22.2)	13 (6.2)	
**Combination treatment methods**	Whole-brain radiation therapy	1 (1.4)	3 (3.5)	1 (2.9)	3 (16.7)	8 (3.8)	0.024
	Palliative radiation therapy	1 (1.4)	1 (1.2)	1 (2.9)	0 (0)	3 (1.4)	0.835

Note: *NSCLC-not otherwise specified (NOS): 2 patients, adenosquamous carcinoma: 1 patient. **13 patients had uncommon EGFR mutation (6.2%), L858R and Del19: 1 (0.05%), Del19 and S768I: 1 (0.05%), G715A: 1 (0.05%), L861Q: 1 (0.05%), S768I: 1 (0.05%); G719C and E709A:1 (0.05%), G718D and S768I:1 (0.05%); L858R and T790M: 3 (1.4%), G719C: 3 (1.4%).

### Objective response

The overall ORR and DCR were 83.4% and 95.7% in the Osimertinib group, 73.6% and 97.1% in the Afatinib group, 76.5% and 94.1% in the Gefitinib group, and 77.1% and 97.3% in the Erlotinib group, respectively (Table 2).

**Table 2:** Treatment response according to RECIST 1.1

Characteristic	Erlotinib (n = 74) n (%)	Gefitinib (n = 85) n (%)	Afatinib (n = 34) n (%)	Osimertinib (n = 18) n (%)	Total n (%)	*p*-value
Complete response	1 (1.4)	1 (1.2)	0 (0)	1 (5.5)	3 (1.4)	–
Partial response	56 (75.7)	64 (75.3)	25 (73.6)	14 (77.9)	159 (75.4)	–
Stable disease	15 (20.2)	15 (17.6)	8 (23.5)	2 (11.1)	40 (19)	–
Progressive disease	2 (2.7)	5 (5.9)	1 (2.9)	1 (5.5)	9 (4.3)	–
Objective response rate	57 (77.1)	65 (76.5)	25 (73.6)	15 (83.4)	162 (76.7)	0.887
ORR (CNS)	14 (66.7)	15 (68.2)	5 (71.4)	6 (85.7)	40 (70.2)	0.807
Disease control rate	72 (97.3)	80 (94.1)	33 (97.1)	17 (94.5)	202 (95.7)	0.752

**Figure 2:**
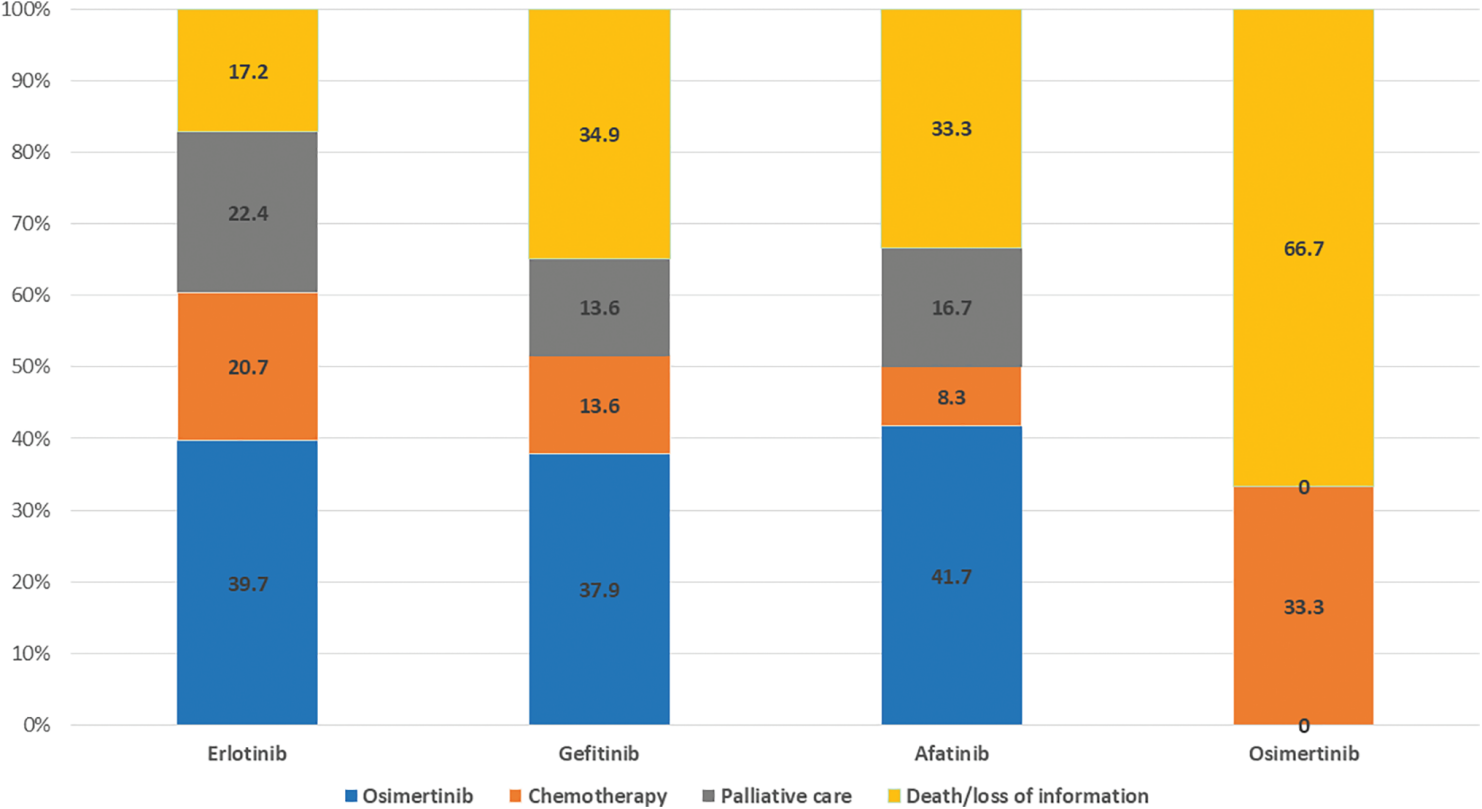
Subsequent therapies in patients who discontinued first-line.

At the time of data cutoff, 142 patients (67.3%) discontinued first-line treatment. Patients receiving second-line treatment with Osimertinib after failing first-line treatment with generations I and II TKIs accounted for 37.9%–41.7% of each drug group. Among the 6 patients who progressed after Osimertinib treatment, 2 (33.3%) switched to chemotherapy. Patients who switched to palliative care in the Erlotinib, Gefitinib, and Afatinib groups were 22.4%, 13.6%, and 16.7%, respectively (Fig. 2).

Illustration of patterns of treatment switching from first-line to second-line treatment. Blue, orange, brown, and yellow represent second-line treatment options such as osimertinib, chemotherapy, palliative care, and death/loss of information, respectively. Numbers in the figure indicate percentages in each group.

**Figure 3:**
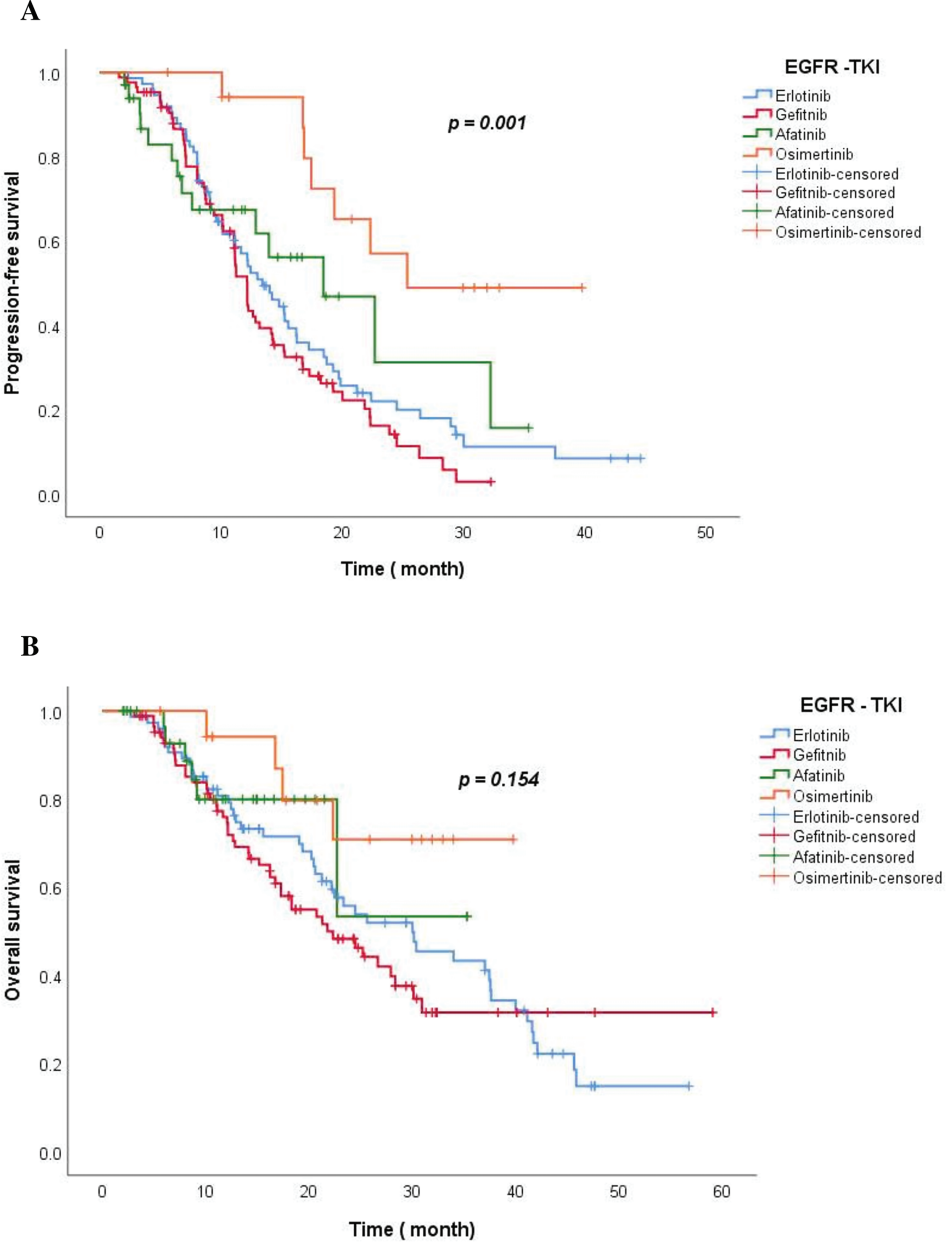
The Kaplan-Meier curve of PFS (A) and OS (B) for TKI drug groups.

At the time of analysis on 30 August 2023, the median follow-up time was 42.1 months in the Erlotinib group, 28.4 months in the Gefitinib group, 11.9 months in the Afatinib group, and 29.9 months in the Osimertinib group. The median PFS of Osimertinib was longest at 25.3 months, Afatinib was 18.4 months (95% CI: 10.1–26.8); Erlotinib was 13.4 months (95% CI: 10.6–16.2) and Gefitinib was the lowest at 12.2 months (95% CI: 11.1–13.2) (*p* = 0.001) (Fig. 3).

The median OS for the Osimertinib and Afatinib group was not reached; 30.0 months with the Erlotinib group (95% Cl: 19.1–40.9) and 21.8 months with the Gefitinib group (95% Cl: 15.0–28.4) (*p* = 0.154). At 12 months, the estimated percentage of patients who were alive was 74.5% in the Gefitinib group, 79.2% in the Erlotinib group, 79.9% in the Afatinib group, and 94.1% in the Osimertinib group. At 4 years, 14.7% of the patients remained alive in the Erlotinib group (Fig. 3).

Blue, red, green, and orange represent the TKIs Erlotinb, Gefitinib, Afatinib, and Osimertinib, respectively.

Assessing simultaneously the relationship between multiple risk factors and patients’ PFS using the Cox model demonstrated that Osimertinib was more effective than first-generation TKIs (Erlotinib and Gefitinib) in patients with Del 19 mutations [*p* = 0.019; HR = 0.37, (95% CI: 0.15–0.85)], in patients without brain metastases [*p* = 0.004; HR = 0.13 (95% CI: 0.03–0.52)], and in patients with PS ≥ 2 [*p* = 0.005; HR = 0.13 (95% CI: 0.03–0.54)], in patients <65 years [*p* = 0.016; HR = 0.29 (95% CI: 0.10–0.79)] and ≥ 65 years [*p* = 0.022; HR = 0.25 (95% CI:0.08–0.81)], in male patients [*p* = 0.041; HR = 0.29 (95% CI:0.09–0.95)] and female patients [*p* = 0.006; HR = 0.24 (95% CI: 0.08–0.66)], and in non-smoker patients [*p* = 0.005; HR = 0.23 (95% CI: 0.08–0.63)]. Afatinib and Gefitinib had no difference in PFS compared to Erlotinib in subgroups with or without brain metastases, del 19 or L858R mutation, PS < 2 or PS ≥ 2, age ≥65 or age <65, male or female, non-smoker or smoker (*p* > 0.05) (Table 3).

**Table 3:** Relationship of progression-free survival time with characteristic factors

Characteristic	HR (95% CI)	*p*
Del 19: 1st generation TKI (erlotinib and gefitinib) (reference)		
Afatinib	0.78 (0.37–1.62)	0.502
Osimertinib	0.37 (0.15–0.85)	0.019
L858R: 1st generation TKI (erlotinib and gefitinib) (reference)		
Afatinib	0.42 (0.12–1.34)	0.141
Osimertinib	0.24 (0.03–1.72)	0.154
CNS metastases: 1st generation TKI (erlotinib and gefitinib) (reference)		
Afatinib	2.38 (0.70–8.05)	0.162
Osimertinib	0.42 (0.16–1.09)	0.074
No CNS Metastases: 1st generation TKI (erlotinib and gefitinib) (reference)		
Afatinib	0.62 (0.33–1.17)	0.141
Osimertinib	0.13 (0.03–0.52)	0.004
PS < 2: 1st generation TKI (erlotinib and gefitinib) (reference)		
Afatinib	0.66 (0.35–1.20)	0.173
Osimertinib	0.43 (0.17–1.05)	0.065
PS ≥ 2: 1st generation TKI (erlotinib and gefitinib) (reference)		
Afatinib	2.75 (0.64–11.84)	0.174
Osimertinib	0.13 (0.03–0.54)	0.005
Age < 65: 1st generation TKI (erlotinib and gefitinib) (reference)		
Afatinib	0.05 (1.48–2.32)	0.892
Osimertinib	0.29 (0.10–0.79)	0.016
Age ≥ 65: 1st generation TKI (erlotinib and gefitinib) (reference)		
Afatinib	0.57 (0.26–1.26)	0.167
Osimertinib	0.25 (0.08–0.81)	0.022
Male: 1st generation TKI (erlotinib and gefitinib) (reference)		
Afatinib	0.51 (0.24–1.09)	0.082
Osimertinib	0.29 (0.09–0.95)	0.041
Female: 1st generation TKI (erlotinib and gefitinib) (reference)		
Afatinib	0.93 (0.37–2.33)	0.886
Osimertinib	0.24 (0.08–0.66)	0.006
Nonsmoker: 1st generation TKI (erlotinib and gefitinib) (reference)		
Afatinib	0.85 (0.34–2.12)	0.734
Osimertinib	0.23 (0.08–0.63)	0.005
Smoker: 1st generation TKI (erlotinib and gefitinib) (reference)		
Afatinib	0.55 (0.26–1.19)	0.132
Osimertinib	0.31 (0.09–1.31)	0.052

### Adverse events

The most common AEs were diarrhea, skin rash, and paronychia, primarily in grades 1 and 2. Diarrhea was most frequently observed in the Afatinib group at 44.1% (*p* = 0.03); while skin rash was most prevalent in the erlotinib group at 60.8%; the lowest incidence was in the osimertinib group (11.1%) (*p* = 0.003). Paronychia (31.8%) and increased Aspartate transaminase/Alanine transaminase (AST/ALT) levels (27.1%) were more common in the gefitinib group. Interstitial lung disease (ILD) was observed at a very low rate in the treatment groups, with no cases of grades 3–4. Grades 3–4 AEs were most frequent in the afatinib group, with diarrhea (11.8%) and stomatitis (11.8%) being the most common (Table 4).

**Table 4:** Common adverse events (AEs)

AEs	Erlotinib (n = 74)				Gefitinib (n = 85)				Afatinib (n = 34)				Osimertinib (n = 18)				*p*-value (all AEs)
	All		Grades 3–4		All		Grades 3–4		All		Grades 3–4		All		Grades 3–4		
	N (%)				N (%)				N (%)				N (%)				
Diarrhea	21	28.4	3	4.1	17	20	4	4.7	15	44.1	4	11.8	0	0	0	0	0.003
Paronychia	9	12.2	0	0	27	31.8	0	0	4	11.8	0	0	1	5.6	0	0	0.003
Skin rash	45	60.8	7	9.5	40	47.1	2	2.4	12	35.3	2	5.9	2	11.1	0	0	0.001
Stomatitis	13	17.6	1	1.4	10	11.8	0	0	6	17.6	4	11.8	0	0	0	0	0.212
ILD	1	1.4	0	0	3	3.5	2	2.4	1	2.9	0	0	0	0	0	0	0.727
AST/ALT increased	12	16.2	1	1.4	23	27.1	3	3.5	1	2.9	0	0	0	0	0	0	0.002

Note: Statistical significance was set at *p* < 0.05. ILD: Interstitial lung disease. AST: Aspartate transaminase. ALT: Alanine transaminase.

## Discussion

The mean age of patients in the Afatinib (68.1 years) and Osimertinib (67.4 years) arms was higher than that in the Erlotinib (62.3 years) and Gefitinib (64.3 years) arms. Most young patients are typically the main breadwinners in their families, so illness significantly affects their finances and ability to cover medical expenses. Meanwhile, older patients are often supported financially by their children. Therefore, younger patients tend to prioritize using first-generation TKIs with lower costs, while older patients choose expensive drugs. The female or non-smoker group was generally higher than the male or smoker group, respectively. However, in the Afatinib group, the male group accounted for a higher proportion (61.8%), which resulted in a higher smoking group (55.9%) in the Afatinib group than non-smokers (44.1%). Patients who had an ECOG PS 0–1 were in the majority with 73.9%. We found that patients in the Osimertinib group had a worse performance status, with the proportion of patients having an ECOG PS ≥ 2 accounting for 38.9%. Osimertinib has proven safer than 1st and 2nd generation TKIs in clinical trials, therefore, it is often preferred for this group of patients [9]. In contrast, with data in clinical trials, the toxicity of Afatinib is often higher than that of other TKIs, so in the Afatinib group, the overall performance status was better, with only 11.8% of patients having an ECOG PS ≥ 2 [20]. Our study included 57 patients (27%) who were found to have brain metastases at the time of diagnosis. The Osimertinib treatment group had the highest rate of brain metastases (38.9%). According to preclinical data, Osimertinib shows significantly better brain penetration than first and second-generation EGFR-TKIs [21]. In addition, the FLAURA trial demonstrated a consistent benefit of Osimertinib compared with first-generation TKIs in patients with brain metastases. Therefore, Osimertinib is often preferred in cases of brain metastases.

The overall response rate was 76.7%, of which the ORR of Osimertinib was the highest (83.4%). At the end of the study, 142 patients (67.3%) failed first-line treatment. The group that continued first-line therapy included 69 patients (32.7%), comprising 19 patients (22.4%) in the Gefitinib, 16 patients (21.6%) in the Erlotinib, 22 patients (64.7%) in the Afatinib, and 12 patients (66.7%) in the Osimertinib. Subsequent treatment options include Osimertinib, chemotherapy, and palliative care. Patients treated with Osimertinib after failure of first-line with 1st and 2nd generation TKIs accounted for 37.9%–41.7%, of which patients starting treatment with Afatinib had the highest rate of treatment, followed by Osimertinib. When the disease is progressive, we prioritize performing a solid biopsy to re-evaluate the histopathology, searching for the T790M mutation. In cases where the patient’s physical condition is not suitable, there are underlying illness that contraindicate biopsy, or the biopsy site is challenging, we conduct a liquid biopsy. However, not all patients are tested for the T790M mutation and have the opportunity to use 3rd generation TKIs. First, some patients with poor physical condition and severe underlying diseases are referred to palliative care. Second, T790M mutation testing is currently not covered by health insurance, and there is a significant rate of false negatives, especially with liquid biopsy. Finally, the price of Osimertinib remains very high compared to the income of Vietnamese patients. Although there are many barriers to T790M testing and access to Osimertinib treatment, our second-line treatment rate with Osimertinib aligns with several other studies, ranging from 24.5% to 43.1% [18,22]. Many studies have shown that sequential treatment with Afatinib and Osimertinib is a feasible and effective treatment strategy in Asian patients, helping to prolong the chemotherapy-free time by more than 3 years and achieving an OS of nearly 4 years [23,24].

In our study, Osimertinib’s median PFS was the longest at 25.3 months; Afatinib was 18.4 months (95% CI: 10.1–26.8); Erlotinib was 13.4 months (95% CI: 10.6–16.2) and the lowest was Gefitinib with 12.2 months (95% CI: 11.1–13.2) (*p* = 0.001). This result is similar to that of clinical trials of first-generation TKI drugs, which reported PFS from 9–13 months, twice as long as chemotherapy [6,8]. The median PFS of the Afatinib and Osimertinib arms was significantly longer than that in clinical trials, such as LUX LUNG 7 [20] at 11 months and FLAURA [9] at 18.9 months, but was similar to that of real-world studies in Korea, China, Taiwan, Japan... Some real-world studies in Korea, such as the study by author Lee Sung Yong on 422 patients treated with Afatinib, reported a time to treatment failure was 17.8 months [18]. A study by Youjin Kim et al. on 467 NSCLC patients with EGFR mutations treated with first-line TKIs at Samsung Medical Center from 2014 to 2016 showed median PFS for Afatinib, Gefitinib, and Erlotinib were 19.1 months, 13.7 months, and 14.0 months, respectively [25]. A real-world study by Sakata et al. in Japan involving 538 patients treated with first-line Osimertinib reported a PFS of 20.5 months [26]. Another Japanese study by Uehara et al. involving 485 patients showed that the PFS of first-line Osimertinib was 23.4 months, while that of first and second-generation TKIs was 13.9 months [27]. The median PFS time for each TKI in our study was longer than that reported in prospective trials such as NEJ002 [28], OPTIMAL [6], LUXLUNG 3 [7], and FLAURA [9]. This may be because real-world studies often evaluate more flexibly, and with practical clinical experience, the assessment and management of AEs are more effective than before, a smaller number of patients (Osimertinib group n = 18) may partly contribute to longer PFS. Additionally, the incidence of grade 3 or 4 AEs with Osimertinib, Afatinib, Gefitinib, and Erlotinib in our study was lower than that reported in previous clinical trials.

The median OS for the Osimertinib and Afatinib arms in our study was not reached; 30.0 months (95% Cl: 19.1–40.9) in the Erlotinib group and 21.8 months (95% Cl: 15.0–28.4) in the Gefitinib group (*p* = 0.154). Because Afatinib and Osimertinib were licensed in Vietnam after the first-generation TKIs, the number of patients was limited, and the follow-up time for patients in these groups was not long enough. After 1 year, 74.5% of the patients remained alive in the Gefitinib group, 79.2% in the Erlotinib group, 79.9% in the Afatinib group, and the highest was 94.1% in the Osimertinib group. At 2 years, 48.2%, 54.0%, 53.3%, and 70.8% of the patients were alive in the Gefitinib, the Erlotinib, the Afatinib, and the Osimertinib arms, respectively. According to the FLAURA study, the OS of the Osimertinib group was 38.6 months compared to 31.8 months for the Gefitinib/Erlotinib group; after 1 year, 83% of patients in the 1st generation TKI group were alive, 89% in the Osimertinib group; after 2 years, it was 59% in the 1st generation TKI group and 74% in the Osimertinib group [9].

Osimertinib was more effective than first generation TKIs (Erlotinib and Gefitinib) in the group of patients without CNS metastases [*p* = 0.004; HR = 0.13 (95% CI: 0.03–0.52)], Del 19 gene mutation [*p* = 0.019; HR = 0.37, (95% CI: 0.15–0.85)], PS ≥ 2 [*p* = 0.005; HR = 0.13 (95% CI: 0.03–0.54)], <65 years [*p* = 0.016; HR = 0.29 (95% CI: 0.10–0.79)] and ≥65 years [*p* = 0.022; HR = 0.25 (95% CI: 0.08–0.81)], male [*p* = 0.041; HR = 0.29 (95% CI: 0.09–0.95)] and female [*p* = 0.006; HR = 0.24 (95% CI: 0.08–0.66)], and in non-smoker [*p* = 0.005; HR = 0.23 (95% CI: 0.08–0.63)]. In the group of patients with CNS metastases, L858R, PS < 2, and smoker, Osimertinib tended to improve PFS; however, the difference was not statistically significant. This result differs from that of the FLAURA trial, in which Osimertinib improved PFS in all subgroups, possibly due to the limited sample size of the Osimertinib group, which provided insufficient data for the analysis [9]. In the Osimertinib group, 3/7 (42.8%) of patients received whole-brain radiation for multifocal brain tumors with central nervous system symptoms, which was higher than that in the first-generation TKI group (3/43 [7%]). Additionally, the central nervous system response rate in the Osimertinib group was the highest (85.7%) compared with the other TKIs. This is a promising result; however, further studies with larger patient numbers and longer follow-up periods are needed to clarify the effectiveness of Osimertinib compared with other TKIs. Across the Afatinib groups, no statistically significant differences were observed between the patient groups treated with Erlotinib and those treated with Gefitinib. A real-world study by Kim et al. of 467 patients treated with first-line TKIs (Afatinib, Gefitinib, or Erlotinib) showed no difference in the L858R subgroup between TKIs [25].

The treatment goal in the advanced stages of NSCLC is to improve survival and quality of life. Therefore, treatment-related AEs should always be considered when selecting treatment methods for patients. The most common AEs were diarrhea, skin rash, and paronychia, mostly in grades 1 and 2. Diarrhea was most frequently observed in the Afatinib at 44.1% (*p* = 0.03), while increased AST/ALT levels (27.1%) were more common in the gefitinib than in the other groups (*p* = 0.002). This result is similar to the analysis of the TKI drug safety profile by the US Food and Drug Administration [29]. Skin rash was most common in the erlotinib at 60.8%, while the lowest incidence was found in the osimertinib (11.1%) (*p* = 0.003). Paranychia (31.8%) was highest in the gefitinib (*p* = 0.001). Our findings differ from the meta-analysis by Yi Zhao et al., where Afatinib was identified as the drug with the highest adverse effect [30]. Interstitial lung disease (ILD) was observed at a very low rate in the treatment groups, with no cases of grades 3–4. High-grade AEs (grades 3–4) were most frequent in patients treated with Afatinib, with diarrhea (11.8%) and stomatitis (11.8%) being the most common (Table 4). Compared with previous clinical trials, the incidence of AEs was lower, and no new toxicities were observed [6,9,20]. Thus, TKIs for advanced stage NSCLC patients treatment is safe and rarely causes serious toxicity.

This is the first and largest real-world study involving 211 patients to comprehensively evaluate patient characteristics and the effectiveness of TKIs in Vietnam, with long follow-up from January 2017 to August 2023. This study provides a thorough overview of the treatment results of all three generations of TKIs, as well as the differences in effectiveness between drugs in each patient subgroup.

Our study has some limitations. Because of its retrospective nature, the results are biased due to differences in patient characteristics between treatment groups. Second, Afatinib and Osimertinib were licensed by the Ministry of Health later, leading to inadequate OS due to a short follow-up time. Finally, there are differences in TKI drug prices and coverage by Vietnamese health insurance (1st and 2nd generation TKIs are covered by 50%, while 3rd generation is not covered). After deducting the subsidies, the amount patients pay for third-generation TKIs and second-generation drugs is 322% and 28% higher, respectively, than for first-generation drugs. The price of Osimertinib is 4.5 times Vietnam’s per capita income.

## Conclusion

This study provides comprehensive information on the diagnosis and treatment of NSCLC patients with EGFR mutations and the outcome of first-line TKI therapy in these patients in a real-world setting in Nghe An, Vietnam. Our study demonstrated that TKIs have benefits in terms of the overall response rate, prolonged survival time, and enhanced tolerability.

## Data Availability

The datasets used or analyzed during the current study are available from the corresponding author upon reasonable request.
